# A measure for quantifying the impact of housing quality on respiratory health: a cross-sectional study

**DOI:** 10.1186/1476-069X-11-33

**Published:** 2012-05-14

**Authors:** Michael D Keall, Julian Crane, Michael G Baker, Kristin Wickens, Philippa Howden-Chapman, Malcolm Cunningham

**Affiliations:** 1 He Kainga Oranga/Housing and Health Research Programme, Department of Public Health, University of Otago, PO Box 7343, Wellington South, New Zealand; 2 Wellington Asthma Research Group, Department of Medicine, University of Otago, Wellington, New Zealand; 3 Building Research Association New Zealand, Porirua City, New Zealand

**Keywords:** Respiratory health, Home environment, Asthma symptoms

## Abstract

**Background:**

Damp and mould in homes have been established as risk factors for respiratory health. There is a need for a relatively straightforward assessment of the home that quantifies this risk.

**Methods:**

Using data from 891 New Zealand houses, the utility of a Respiratory Hazard Index quantifying key attributes related to damp and mould was tested by studying its associations with self-reported respiratory symptoms.

**Results:**

A dose–response relationship was found whereby each unit increase in the Respiratory Hazard Index was associated with an 11% increase in the odds of at least one episode of wheezing/whistling in the chest over the last 12 months (relative odds of 1.11 with a 95% CI 1.04%–1.20%). An 11% increase in the odds of an asthma attack over the last 12 months was estimated (relative odds of 1.11 with a 95% CI 1.01%–1.22%). These estimates were adjusted for household crowding levels, age, sex and smoking status. There was suggestive evidence of more steeply increasing odds of respiratory symptoms with increasing levels of the Respiratory Hazard Index for children aged under 7. In the worst performing houses according to the Index, a 33% reduction in the number of people experiencing respiratory symptoms (relative risk 0.67 with 95% CI 0.53 to 0.85) could be expected if people were housed in the best performing houses.

**Conclusions:**

This study showed that increased evidence of housing conditions supporting dampness and mould was associated with increased odds of respiratory symptoms. A valid housing assessment tool can provide a rational basis for investment in improved housing quality to improve respiratory health.

## Background

There is growing awareness of the importance of neighbourhood, indoor environment, housing and occupant behaviour and lifestyles to health in general[1] and respiratory health in particular[2]. The recognition that we spend an increasing amount of our lives indoors [3] has encouraged research interest in indoor air quality and the indoor environment in general. Broadly these risk factors for poor indoor air quality may be conceptualised as arising mainly from the ‘building determinants’ (the building envelope, construction and maintenance), and ‘household factors’ (behaviour and lifestyle of occupants) along with a small contribution from neighbourhood environment.

Building-related moisture damage and microbial growth may increase the risk of asthma, as well as other respiratory disorders [4]. Andriessen et al. (1998) found an association between self-reported moulds in homes and both objective (e.g., peak flow variability) and subjective (e.g., frequency of respiratory symptoms) markers of airway lability in European children with chronic respiratory symptoms [5]. Moreover, other studies have reported associations between dampness, mould and asthma among children [6,7]. A recent randomised controlled trial involving mould removal and improved home ventilation resulted in improved asthma symptoms, indicating a causal link between the presence of mould and damp, and the symptoms [8]. The mechanism for these associations is unclear. A small component is due to direct sensitisation to mould but mould sensitisation is generally uncommon. However, dampness is also associated with mould and bacterial growth and both mould and bacterial products (β glucans and endotoxin respectively) have recently been shown to be associated with wheezing in adults [9] and in infants.[10] An association between endotoxin exposure and wheezing in the first 15 months of life has been found in a cohort of NZ children[11]. A further possible link in the associations between respiratory health and dampness may be viral. Most exacerbations of asthma are associated with viral respiratory tract infection [12], most commonly in winter, when housing conditions tend to be damper.

This evidence is particularly pertinent to New Zealand. Two thirds of New Zealand houses are timber framed on concrete or wooden piles with iron roofs, and a third are uninsulated. Surveys of occupants in New Zealand homes[13] revealed 35% reporting visible mould in one or more living rooms or bedrooms. New Zealand also has one of the highest asthma rates in the world [14], to which poor housing quality is likely to contribute.

New Zealand housing has been the focus of two major randomised controlled trials that examined the effects of housing improvements on the respiratory health of asthmatic occupants. Insulating existing houses led to a significantly warmer, drier indoor environment, with improved self-rated health, days off school and work, and visits to general practitioners [15]. Installing non-polluting, effective home heating in the households of asthmatic children led to a reduction in symptoms of asthma, improved wellbeing, and fewer days off school [16]. These studies demonstrated clear respiratory health benefits of warm and dry housing.

In most established towns and cities, the residential housing stock is largely already built, so improving the quality of the built environment to improve occupant health would benefit from diagnostic tools that can identify deficiencies in the indoor environment that could be addressed via retrofitted improvements to the building [17]. The Healthy Housing Index is a housing quality assessment protocol administered by trained building inspectors. It was developed to inform research into links between housing quality and health with a focus on issues that may be particularly salient in New Zealand [18]. The injury hazard component of the Healthy Housing Index has been shown to be significantly correlated with the risk of injuries to the occupants of the house [19]. As the Healthy Housing Index is also intended to be used as a practical rating tool to assess housing quality, the measurements are deliberately non-invasive and do not involve damage to the building fabric.

This paper describes the Respiratory Hazard Index developed from measurements made of the home environment together with an analysis of associations between levels of this Index and self-reported respiratory symptoms from a sample of households in the Taranaki Region of New Zealand. Taranaki is a region in the west of the North Island of New Zealand with a 2006 population of 104,127 [20]. The climate is temperate, ranging between monthly averages of 10 and 18 degrees Celsius in the main city (New Plymouth) in July and January respectively, with a relatively high annual rainfall of 1,432 mm.

## Methods

### Subjects

A sample was taken of 891 Taranaki homes built before 1980 occupied by at least one pensioner, unemployed person, sickness beneficiary or student. The sample was recruited over the period August 2007 to September 2008 from households applying to have insulation retrofitted to their houses under a scheme that provided a 100% subsidy for the costs.

### Data

The participants were asked to self-report respiratory symptoms, current smoking behaviour and use of medication for asthma. Trained inspectors, who were blind to the disease status of the occupants, assessed the homes using procedures developed during the Healthy Housing Index Pilot Study [18]. The assessment of each home took about an hour and involved a walk-through of all the rooms in the house and completion of a checklist to identify features of the house that had potential health and safety effects. Some of the measurements made were subjective and were a particular focus of the inspectors’ training and peer review. These included judgements as to whether the house “smells musty” and “feels damp”, and assessing the extent of mould visible on walls. The identification of a musty smell is a measure that has been found to have consistent associations with respiratory tract symptoms [21]. The “mould odour” is thought to signal the presence of hidden moulds that are otherwise not detectable. Of the households approached, only 34 were unwilling to participate or could not be contacted, a response rate of 97%. Some houses assessed using the Healthy Housing Index did not have matching questionnaire data for the occupants, and some questionnaire data from occupants could not be matched to housing data because of lost forms. Only households with complete data were retained for the study, resulting in a sample of 907 houses with 2040 occupants. From these, we excluded those with first residence in the house between years 2007 and 2008 due to reduced exposure to the housing conditions evaluated. This ensured that the symptoms reported could be validly related to the housing exposures assessed at the time of recruitment. The final sample consisted of 891 houses with 1756 occupants.

### Formation of the respiratory hazard index (RHI)

The RHI is a count of housing characteristics considered by expert opinion and surveys of the literature to be linked to the respiratory health of the occupants of the house. These items are listed in Table 1 together with their prevalence in the sample. The last two items were not studied as none of the houses had ceiling or wall insulation. Three of the items, “feels quite damp”, “house in shade – substantial” and “major leaks in roof” each contributed two points to the RHI; these are more extreme versions of “feels a little damp”, “house in shade – partial” and “minor leaks in roof” respectively, which each contributed one point to the RHI.

**Table 1:** Components of a housing Respiratory Hazard Index together with proportion of houses with specified hazard as rated by inspector (N = 891; percentages calculated on valid responses only)

**Feature assessed**	**N**	**%**
feels a little damp	182	23%
feels quite damp	32	4%
in shade - partial	389	49%
in shade - substantial	82	10%
house smells musty	37	5%
any mould in bedrooms/living rooms	39	4%
unflued gas heater* in any bedrooms/living rooms	212	24%
fungi/mould on joists or bearers	22	4%
no floor insulation	527	87%
minor leaks in roof	114	13%
major leaks in roof	26	3%
ponding of water under house	32	4%
no ceiling insulation**	891	100%
no wall insulation**	891	100%

*includes unflued reticulated gas heaters as well as portable LPG heaters.

**not included in the Respiratory Hazard Index for the current study as no houses had wall or ceiling insulation at the time of the study.

**Figure 1:**
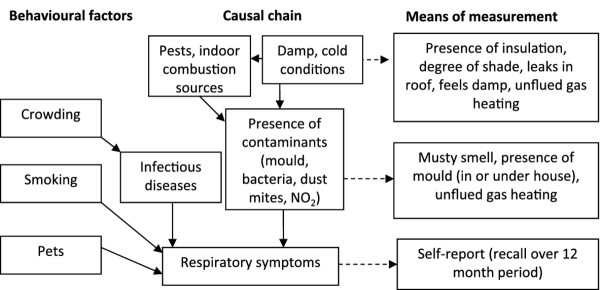
Representation of relationship between causal factors of respiratory symptoms and measurements made in study.

Figure 1 shows a diagram of behavioural and environmental factors affecting respiratory symptoms, together with the measurements made. Damp and cold housing conditions support biocontaminant growth, providing favourable conditions for mould, bacteria and dust mites. Factors associated with damp and cold conditions were assessed, as were visual and olfactory evidence of mould. Unflued gas heating releases both large quantities of moisture and nitrogen dioxide (NO_2_), and so appears in two boxes in Figure 1. Respiratory symptoms were self-reported by the respondents, who were unaware of the results of the housing inspection. We collected self-reported smoking behaviour and house occupancy. Crowding is a well-known factor in the transmission of infectious diseases, which in turn can lead to respiratory symptoms [22]. Rather than use existing crowding indices, which allow for the social acceptability of sharing bedrooms, we assessed close contact exposure to other household members in terms of number of occupants per available bedroom. SES was not accounted for in the analysis, despite its demonstrated association with respiratory health [23] as the sample was uniformly relatively socioeconomically deprived.

### Analysis

Logistic regression was carried out using the presence or absence of given respiratory symptoms over the previous 12 months as the dependent variable, adjusted for household crowding levels, age, sex and smoking status. These self-reported symptoms were: wheezing or whistling in the chest; or an asthma attack. Four regressions were performed to estimate: (i) the odds of wheezing or whistling, with the RHI treated as a categorical variable; (ii) as for the first regression, but with the RHI fitted as a continuous variable (so the effect estimated represents the odds associated with an increase in one level of the RHI); (iii) the odds of an asthma attack, with the RHI treated as a categorical variable; (iv) as for the third regression, but with the RHI fitted as a continuous variable.

## Results

Preliminary data analyses were conducted to look at crude associations between factors and to identify potential threats to the validity of the analysis. Tables 1–2 show characteristics of the surveyed houses and their occupants.

**Table 2:** Self-reported health of occupants in 12 months preceding recruitment - % reporting symptoms or smoking status (N = 1660 occupants)

	**N**	Prevalence*
Influenza	958	60%
Wheezing or whistling in your chest	634	40%
Wakened with shortness of breath	337	21%
Asthma attack	246	15%
Taking asthma medication	327	20%
Do you smoke one or more per day?	202	12%
Wheezing or whistling and/or asthma	655	41%

* amongst those with valid data.

Table 1 shows that the vast majority of houses did not have under-floor insulation and none had ceiling or wall insulation. The next most common characteristic was partial shading of the house from the sun, followed by the presence of unflued gas heating and the subjective sense of dampness. Most houses had more than one of the characteristics listed, with only 15% rated as having no characteristics contributing to the RHI. Almost one quarter (23%) of houses had one characteristic; 29% had two characteristics; 17% had three; 9% had four; 4% had five; and 2% had six or more. The proportion of daily smokers (one or more cigarettes smoked per day) was 12%, considerably lower than the 2006 Census rate of 22% for the area [20]. The reason for this difference is unclear. The second column of Table 2, which shows self-reported health status and respiratory symptoms over a 12-month period preceding recruitment, shows the number of respondents with valid data, which acts as the denominator for the percentage in the last column. Almost 60% of the sample reported an episode of influenza. 20% of the sample reported taking asthma medication, similar to the rate amongst socioeconomically deprived areas found in another study [23]. Table 2 shows that the most common respiratory symptom reported was “wheezing or whistling in the chest”. It was reported by 92% of those also reporting an asthma attack.

**Figure 2:**
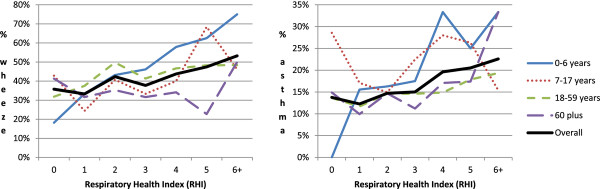
Proportion by age group and overall reporting an episode of wheezing or whistling in the chest (LH graph) or asthma attack (RH graph) during the past 12 months by the housing respiratory hazard index for their home.

Figure 2 shows the crude (unadjusted) proportions by age group who reported symptoms, according to the rating of their home using the RHI. Index levels at 6 and above were combined in this graph to enable a proportion to be calculated for the level “6 and above” (at each level individually the data were too sparse). These graphs show generally rising prevalence overall with an increase in the index, but marked differences between age groups. Although children aged 0–6 showed dramatic increases in the proportion experiencing the respiratory symptoms, the associations between increasing levels of the RHI and reported symptoms were less strong for older age groups.

As shown in Figure 1, there can be expected to be associations between some components of the RHI, particularly those that are part of the same causal chain. The Pearson correlation coefficients in Table 3 show statistically significant correlations bolded. These associations were expected, given that they arise from common conditions. When forming any index, particular aspects can be assigned excessive importance when highly correlated factors are included. The correlations within our data were not large, however: none was greater than 0.17. This suggests that different aspects of the home environment were being evaluated by these different components of the RHI.

**Table 3:** Pearson correlation coefficients for the different components of the RHI measured. Coefficients estimated to be statistically significantly different from 0 are bolded

	**unflued**	**any mould**	**underfloor insulation**	**underfloor fungi**	**a lot in shade**	**a little in shade**	**musty**	**very damp**	**a little damp**	**major leaks**	**minor leaks**	**ponding**
unflued	1.00	0.06	**0.11**	0.00	**0.07**	**0.08**	0.04	−0.01	0.02	−0.02	0.00	0.01
any mould	0.06	1.00	0.01	**0.11**	0.06	**0.07**	**0.09**	**0.11**	0.03	0.06	0.00	0.05
underfloor insulation	**0.11**	0.01	1.00	**0.07**	**0.08**	**0.12**	**0.07**	0.05	**0.12**	**0.09**	0.04	−0.05
underfloor fungi	0.00	**0.11**	**0.07**	1.00	0.02	0.06	0.00	0.05	0.03	0.02	0.03	0.05
a lot in shade	**0.07**	0.06	**0.08**	0.02	1.00	*	**0.07**	0.00	0.05	0.06	−0.03	−0.02
a little in shade	**0.08**	**0.07**	**0.12**	0.06	*	1.00	0.03	0.05	0.04	0.06	0.02	−0.04
musty	0.04	**0.09**	**0.07**	0.00	**0.07**	0.03	1.00	**0.17**	**0.15**	**0.10**	**0.09**	−0.01
very damp	−0.01	**0.11**	0.05	0.05	0.00	0.05	**0.17**	1.00	*	**0.18**	0.05	0.03
a little damp	0.02	0.03	**0.12**	0.03	0.05	0.04	**0.15**	*	1.00	0.03	**0.12**	0.05
major leaks	−0.02	0.06	**0.09**	0.02	0.06	0.06	**0.10**	**0.18**	0.03	1.00	*	−0.02
minor leaks	0.00	0.00	0.04	0.03	−0.03	0.02	**0.09**	0.05	**0.12**	*	1.00	0.00
ponding	0.01	0.05	−0.05	0.05	−0.02	−0.04	−0.01	0.03	0.05	−0.02	0.00	1.00

*These coefficients are not calculated as they are artefactual of the measurement (viz., the same house cannot be both “a little” and “a lot”/“very” at the same time).

One of the regressions fitted used indicator variables for the various levels of the RHI. The coefficients estimated from this regression were not inconsistent with an assumption of a linear increase given expected variation around a linear trend (see Table 4). Consistent with this assumption, the coefficients for the other explanatory variables did not change by more than 3% once the RHI was fitted as a linear term. By fitting the same model specification, but with a log rather than a logistic link function (using log-binomial regression), there was estimated to be an increase of 7% in the risk of whistling or wheezing (relative risk 1.07 with 95% CI 1.03–1.11) in a house with a given level of the RHI relative to a house rated one level lower. This means that in the poorer performing houses – those with a count of six according to the RSI – the number of occupants who experienced respiratory symptoms could be expected to be reduced by 33% (relative risk 0.67 with 95% CI 0.53 to 0.85) if these people were housed in the best performing houses, with a count of zero on the RSI.

**Table 4:** Crude odds ratios and adjusted odds ratios (with 95% confidence intervals in brackets) for whistling or wheezing in chest/asthma attack anytime in the last 12 months

**Effect**	**Whistling or wheezing**		**Asthma attack**	
	**Crude odds**	Adjusted* odds	**Crude odds**	Adjusted* odds
Smoke Cigarettes	1.99 (1.46,2.70)	1.94 (1.39,2.71)	0.85 (0.55,1.32)	0.92 (0.58,1.46)
age 0–6 vs 18–59	1.00 (0.71,1.41)	1.34 (0.92,1.93)	1.35 (0.86,2.12)	1.47 (0.91,2.37)
age 7–17 vs 18-59	0.85 (0.62,1.15)	1.08 (0.78,1.51)	1.53 (1.03,2.26)	1.61 (1.06,2.43)
age 60 plus vs 18-59	0.66 (0.52,0.84)	0.63 (0.47,0.85)	0.93 (0.66,1.30)	0.80 (0.55,1.17)
Sex Female vs Male	1.11 (0.90,1.36)	1.12 (0.90,1.39)	0.96 (0.73,1.27)	0.99 (0.75,1.31)
RHI continuous (per increase of 1 level)	1.12 (1.05,1.20)	1.11 (1.04,1.20)	1.13 (1.03,1.23)	1.11 (1.01,1.22)
RHI 1 vs 0	0.87 (0.55,1.38)	0.90 (0.57,1.43)	0.79 (0.42,1.49)	0.77 (0.41,1.44)
RHI 2 vs 0	1.36 (0.87,2.11)	1.44 (0.92,2.25)	1.03 (0.56,1.88)	1.02 (0.56,1.87)
RHI 3 vs 0	1.07 (0.67,1.70)	1.08 (0.67,1.73)	1.03 (0.55,1.93)	0.95 (0.50,1.79)
RHI 4 vs 0	1.46 (0.88,2.45)	1.64 (0.97,2.75)	1.49 (0.76,2.92)	1.46 (0.74,2.88)
RHI 5 vs 0	1.59 (0.87,2.89)	1.21 (0.65,2.25)	1.50 (0.69,3.25)	1.26 (0.56,2.81)
RHI 6 plus vs 0	1.82 (0.94,3.51)	2.04 (1.03,4.06)	1.39 (0.59,3.27)	1.38 (0.58,3.30)
person/bedroom >1 vs < =1	0.93 (0.75,1.14)	0.68 (0.52,0.89)	0.96 (0.73,1.28)	0.70 (0.49,0.99)

*For all factors (apart from discrete levels of the RHI), the factors were adjusted for a continuous RHI; the other factors controlled for are those listed in the first column of the table.

As the data were clustered by household, with all household members exposed to the same housing factors, we allowed for overdispersion in the model. The third and fourth regressions indicated non-significant levels of underdispersion, which we did not account for in the models, leading to somewhat conservative confidence intervals. Accordingly, for the models 1 and 2, the covariance matrix was multiplied by the heterogeneity factors (Pearson Chi-Square/degrees of freedom) of 1.06 and 1.09 respectively; no heterogeneity factors were applied for models 3 and 4. There was no evidence of poor fits, as indicated by statistically non-significant Hosmer-Lemeshow Goodness-of-Fit tests.

To test for any differences between age groups in terms of the relationship between the respiratory symptoms and levels of the RHI, an interaction term between age and the RHI was tested in the models. This interaction term was not statistically significant, although it approached significance (*p* = 0.06) in model 2 (in which the dependent variable was reported wheeze and the RHI was fitted as a continuous variable).

## Discussion

This study aimed firstly to form a measure of the healthiness of a house with respect to respiratory symptoms and secondly look at associations between this measure and reported respiratory symptoms in a sample of houses and their occupants. We firstly defined our respiratory hazard index (RHI) as a sum of indicators of several key observations of housing quality that were linked by prior research to respiratory health. We found a clear dose–response relationship whereby each additional hazard found was associated with an increase in the odds of reported whistling or wheeze amongst the occupants of 11% (relative odds of 1.11, with a 95% CI: 1.04%–1.20%) and similarly for the odds of an asthma attack (relative odds of 1.11, with a 95% CI 1.01–1.22). There was suggestive evidence of more steeply increasing odds with increasing levels of the RHI for children aged under 7.

The strongest relationships found in this study were between self-reported (or parental reported, in the case of children) whistling or wheezing and levels of the RSI. Self-reported asthma attacks, the other main outcome analysed here, may be more likely to be unreliable due to well-documented problems in assigning asthma diagnoses [24]. Despite difficulties in defining asthma among young children and older adults, which may have led to some misclassification, we found a significant association between increased levels of the RHI and self-reported asthma attacks in the total population.

As shown by Figure 2, the youngest age group specified (6 years old and under) appeared to have the strongest sensitivity to increasing levels of the RHI, and the association between the reported symptoms and the RHI then weakened with increasing age. The statistical model for wheeze provided weak evidence that such an interaction between age group and the RHI existed (*p* = 0.06), a phenomenon that merits future investigation. Bronchiolitis and wheezing illness in young children is much more common than in older children or adults. As younger children spend more time at home [3], the home environment may also have a stronger influence on their respiratory health. Of course, this study was observational and the respondents may have chosen dwellings with characteristics appropriate to their health status. Such choices are perhaps more likely for older people who may move to smaller but higher quality housing chosen by them or their families in response to declining health.

Another limitation is that not all relevant exposure data related to respiratory health were collected. The housing measurements were restricted to those related to the building itself and did not include occupier behaviours such as ventilating the house (either with mechanical fans or via opening windows) and owning pets. Nor was outdoor air quality taken into account, although it was expected to vary little within the population we studied. Exposure to airborne particulates could have been measured by specific instruments placed in the house for periods of time. This form of measurement was inconsistent with the purpose of the Healthy Housing Index inspection protocol, which was to provide a practical measure of housing quality related to health based on a single visit to the house. Also inconsistent with this purpose was any form of measurement that could damage the house. This ruled out any thorough assessment of water intrusion arising from leaks in the roof or the external wall cladding.

This research has implications for new public health initiatives. There are already some housing rating systems in use that guide potential buyers and renters as to the quality of a potential home. Some countries such as France require a certificate when a dwelling is offered for sale or rent to highlight potential hazards including lead and asbestos^a^. In addition, all European countries now require an Energy Performance Certificate on sale or rent [25]. Given the potentially important contribution of poor housing quality to respiratory health, the rating systems employing components of the RHI could play an important part in guiding housing choices, particularly for those with existing asthma or with young children. If a causative relationship between housing quality and the reported respiratory symptoms were assumed, in the poorer performing houses, those with a count of six respiratory hazards, the number of occupants who experienced respiratory symptoms could be expected to be reduced by 33% (relative risk 0.67 with 95% CI 0.53 to 0.85) if these people were housed in the best performing houses with a count of zero on the RSI. This is a conservative estimate as the effects of ceiling and wall insulation were not included in the analysis because none of the houses studied had this insulation at the time of the study. As the health benefits of ceiling insulation have been well-established in a randomised controlled trial [26], even the best performing homes studied here could be expected to yield significantly better health outcomes for their occupants once ceiling insulation was installed. It has already been noted that asthma prevalence amongst New Zealand adults increases with increasing levels of deprivation [23]. This study provides further evidence that one of the mediating factors is likely to be housing quality.

Although New Zealand was the setting for this study, we suggest that the components of the RHI identified as important exposures for respiratory health have validity for many other settings where housing may be subject to cold and damp conditions. In the UK, the Housing Health and Safety Rating System has been used to quantify the health impacts of cold and damp in houses, and to put a case for remediation of housing to improve these aspects [27]. As with that pilot study, the current study describes a housing assessment tool that can provide a rational basis for investment in improved housing quality to improve respiratory health.

## Conclusions

This study showed that increased evidence of housing conditions supporting dampness and mould was associated with increased odds of respiratory symptoms. The analysis implies that housing assessment can form a rational basis for investment in improved housing quality to improve respiratory health.

## Endnotes

^a^See http://www.cibi.fr/eng_reports.html

## Competing interests

The authors declare that they have no competing interests.

## Authors’ contributions

MK designed and managed the study, conducted the analysis and drafted the paper. MK, MB, PHC and MC collaborated in the design of the Healthy Housing Index for measuring exposure. All authors read and approved the final manuscript.
